# Supplementary material for: Anti-Inflammatory Effects of Minor Cannabinoids CBC, THCV, and CBN in Human Macrophages
Source: Molecules. 2023 Sep 7;28(18):6487. doi: 10.3390/molecules28186487 (PMC10534668; doi:10.3390/molecules28186487)
Supplement: Supplementary file 1 [file molecules-28-06487-s001.zip › molecules-2589108-supplementary.pdf]

Table S1. The information on the primary antibodies

Primary antibody	Manufacture	Cat #	Dilution
Nlrp3	Cell Signaling Technology	15101S	1:1000
Phosoho-NF-kB-p65 (ser536)	Cell Signaling Technology	13346S	1:1000
NFκB-p-65	Santa Cruz Biotechnology	sc-8008	1:200
Phospho-TYK 2 (Tyr-1054/1055)	Cell Signaling Technology	9321S	1:500
TNFα	Cell Signaling Technology	3707	1:500
PANX-1	Cell Signaling Technology	91137S	1:1000
PPARγ	Cell Signaling Technology	2430	1:1000
Cox-2	Cell Signaling Technology	12282	1:1000
Cleaved Caspase-1	Cell Signaling Technology	4199	1:500
cPLA2	Santa Cruz Biotechnology	sc-376618	1:200
CB1R	Santa Cruz Biotechnology	sc-293419	1:200
β-actin	Santa Cruz Biotechnology	sc-47778	1:200
GAPDH	Santa Cruz Biotechnology	sc-32233	1:200
Phospho-STAT-3(Tyr-705)	Santa Cruz Biotechnology	sc-8059	1:200
STAT-3	Santa Cruz Biotechnology	sc-8019	1:200
Pro-Caspase-1	Santa Cruz Biotechnology	sc-392736	1:200
IL-1β	Abcam	ab216995	1:500
β-actin	Abcam	ab8227	1:2000
GAPDH	Cell Signaling Technology	2118	1:1000
α-tubulin	Santa Cruz Biotechnology	sc-8035	1:200
IL-6	Cell Signaling Technology	12153	1:1000
TYK-2	Cell Signaling Technology	14193S	1:1000
Phospho-STAT-1 (S727)	Abcam	ab109461	1:1000
STAT-1	Abcam	ab92506	1:1000

Table S2. The sequence of qRT-PCR primers

IL-6	Forward	GGAGACTTGCCTGGTGAAA
	Reverse	CTGGCTTGTTCCTCACTACTC
COX-2	Forward	TACTGGAAGCCAAGCACTTT
	Reverse	GGACAGCCCTTCACGTTATT
cPLA2	Forward	TTGGGCGTTTCTGGTTCA
	Reverse	CATCATCACTGTCCGAGCTATC
TNF alpha	Forward	CCAGGGACCTCTCTCTAATCA
	Reverse	TCAGCTTGAGGGTTTGCTAC
Pannexin-1(PANX-1)	Forward	GTGTGCAGCATCAAATCAGG
	Reverse	GACACTGAGCAACTGGAAGA
P2X7	Forward	CTTCCGAGAAACAGGCGATAA
	Reverse	TAGGTTGCAGTCCCAGTAGA
ADAR1	Forward	GTCATCAATGGCCGAGAGTT
	Reverse	CTTGGCTTTGGCTTCCTCTA
Nlrp3	Forward	GAAGAGGAGTGGATGGGTTTAC
	Reverse	TCTGCTTCTCACGTACTTTCTG
IL-1β	Forward	CCTTAGGGTAGTGCTAAGAGGA
	Reverse	AAGTGAGTAGGAGAGGTGAGAG
β-ACTIN	Forward	GGCATCCTCACCCTGAAGTA
	Reverse	CACACGCAGCTCATTGTAGAAG
GAPDH	Forward	CAGGAGGCATTGCTGATGAT
	Reverse	GAAGGCTGGGGCTCATTT
